# Subconjunctival filtration in evolution: current evidence on MicroShunt implantation for treating patients with glaucoma

**DOI:** 10.1186/s40662-022-00322-1

**Published:** 2023-03-02

**Authors:** Iqbal Ike K. Ahmed, Omar Sadruddin, Joseph F. Panarelli

**Affiliations:** 1 John Moran Eye Center, University of Utah, Salt Lake City, UT USA; 2 University of Toronto, Toronto, ON Canada; 3 Prism Eye Institute, Mississauga, ON Canada; 4 Santen Inc, Emeryville, CA USA; 5 New York University, New York, NY USA

**Keywords:** Glaucoma, Micro-invasive glaucoma surgery, MicroShunt, Mitomycin C

## Abstract

**Background:**

Although traditional surgical procedures for glaucoma (such as trabeculectomy and tube-shunt implantation) can significantly reduce intraocular pressure (IOP), they are associated with numerous complications, some of which are vision-threatening, or involve prolonged recovery or a highly intensive postoperative course. Micro-invasive glaucoma surgery (MIGS) procedures have shown better safety but reduced efficacy in achieving target IOP. Combinations of these methods have led to the development of subconjunctival micro-invasive procedures with safety comparable to traditional surgery and greater efficacy than minimally invasive methods. This review describes the use of one of these devices, the poly(styrene-*block*-isobutylene-*block*-styrene) (SIBS)-based PreserFlo MicroShunt (Santen, Emeryville, CA), in the surgical treatment of patients with glaucoma.

**Main text:**

The MicroShunt is an 8.5-mm tube made of an inert polymer with no endplate, an internal diameter of 70 μm, and fins intended to prevent peritubular flow and anchor the device within the sclera to prevent proximal migration into the eye. Following ab externo implantation, the tube provides a conduit for flow of aqueous humor from the anterior chamber into the subconjunctival/sub-Tenon space. Clinical trials to date have shown that, when paired with mitomycin C (MMC) treatment, MicroShunt implantation significantly reduced both IOP and the number of glaucoma medications. These IOP-lowering results were found both when surgery was performed alone and with phacoemulsification. The MicroShunt also showed a safety profile comparable to that of traditional filtering surgery.

**Conclusions:**

The MicroShunt and other novel subconjunctival procedures have shown substantial IOP reductions while mitigating hypotony-related complications. MMC, which modulates fibrosis and scarring postoperatively, is essential to surgical success. Randomized, long-term clinical trials will further clarify the role of controlled micro-incisional device-assisted ab externo glaucoma filtering surgery in long-term glaucoma management.

## Background

The clinical approach to glaucoma management has consisted of a stepwise strategy, beginning with medications and/or laser therapy and progressing to surgical intervention, as needed. This traditional approach balances efficacy and safety, honoring the do-no-harm foundation of medical practice by employing safer therapies early in the treatment course and reserving higher risk interventions for eyes in which target intraocular pressure (IOP) cannot be achieved with medical approaches. The strategy is not perfect as poor adherence and its associated risk of progression remain significant barriers to control. Furthermore, chronic exposure to topical medications and their excipient ingredients can lead to ocular surface disease and contribute to lower success rate with subsequent filtering surgery [1]. Traditional glaucoma surgical procedures such as trabeculectomy and tube-shunt implantation can produce significant IOP reductions and achieve low target IOP, but they are associated with many complications, some of which are vision-threatening [2, 3]. Additionally, traditional filtering surgery is associated with an intense postoperative course, often with numerous interventions and a prolonged recovery [2, 3]. Given that some eyes with glaucoma cannot be adequately managed with medications and/or laser therapy, whether because of advanced disease at diagnosis, the need for a low target IOP, intolerance to or inability to administer eye drops, or a combination of these limiting factors, warrants a surgical procedure that delivers significant efficacy with improved safety.

In recent years, numerous novel glaucoma procedures—collectively termed micro-invasive glaucoma surgeries (MIGS)—have been developed to help meet this unmet need. Initially, these procedures sought to facilitate aqueous humor outflow across the diseased trabecular meshwork and into Schlemm canal, or to bypass the trabecular outflow pathway entirely and utilize the supraciliary space for aqueous drainage. This family of procedures has consistently demonstrated a favorable safety profile with modest efficacy [4–7]. As such, these procedures are typically deployed earlier in the glaucoma disease process, targeting eyes with mild-to-moderate glaucoma. Although MIGS procedures can be performed alone, without phacoemulsification, several of these procedures require combination with cataract surgery, limiting their utility in pseudophakic and non-cataractous eyes.

On this basis, surgical innovation in glaucoma has sought to hybridize the filtration route of traditional procedures with a “MIGS-inspired” minimally invasive technique to produce a subconjunctival filtering procedure that improves upon the safety profile of trabeculectomy while delivering comparable IOP reductions for eyes with more significant disease that require greater efficacy than can be achieved with trabecular or supraciliary MIGS procedures. Consistent with the foundational characteristic of MIGS procedures [8], these novel subconjunctival micro-incisional filtration devices should provide a more consistent surgical procedure than trabeculectomy, while offering faster visual rehabilitation, rendering them appropriate for pairing with elective cataract surgery in phakic eyes or as standalone procedures in pseudophakic or non-cataractous eyes. These have been termed micro-invasive bleb surgeries.

To date, two micro-/minimally-invasive subconjunctival procedures have gained regulatory clearance in various global markets. One is the gel stent (Xen45 Gel Stent implant; Allergan, Dublin, CA, USA), and the other is the poly(styrene-*block*-isobutylene-*block*-styrene) (SIBS)-based PreserFlo MicroShunt (Santen, Emeryville, CA). This review will focus on key studies which furthered the development of the latter device, and the role of the MicroShunt in bridging the gap between MIGS and filtering surgery in the surgical management of glaucoma.

## Main text

### Development and design of the MicroShunt

The MicroShunt is a tube composed of SIBS [9, 10], an oxidatively, hydrolytically, and enzymatically stable material with high tensile strength and high abrasion resistance [9], which incites minimal foreign body reaction in the corneal stroma, under the conjunctiva, or under Tenon’s capsule [11]. The design of the device evolved through three iterations. The first design, the minimally invasive drainage implant (MIDI) tube, featured an 11-mm long tube with a 70-µm lumen diameter, selected to exceed the diameter of a sloughed corneal endothelial cell while providing sufficient resistance to minimize hypotony [10], and a fixation tab extending from the side of the tube at mid-length. In the first-in-human Bordeaux I study in France, 24 eyes with advanced glaucoma, approximately half of which had previously failed trabeculectomy, underwent MIDI-tube implantation without antimetabolite augmentation [10]. This device showed a qualified success rate, defined as IOP ≤ 21 mmHg with a reduction from baseline of ≥ 20% with or without glaucoma medication and with no further incisional procedure, of 42% at 1 year, with two eyes experiencing conjunctival erosion related to the corner of the fixation tab. Before these erosions were noted, the Bordeaux II study was initiated in 16 eyes with advanced glaucoma, 11 of which had failed prior incisional procedures, and incorporated the use of mitomycin C (MMC) to inhibit fibroblast proliferation. Approximately 0.6 mL of a 0.2 mg/mL MMC solution was applied using surgical sponges for 2–3 min, which boosted the 1-year success rate to 67%. At the same time as the Bordeaux II trial, an alternate design consisting of a 12-mm long tube with a 100-µm inner luminal diameter and lacking a fixation tab but having a 7-mm SIBS plate to obviate the need for MMC was evaluated in 12 eyes in the Dominican Republic (the DR I study) [10]. This device resulted in the production of focal cystic blebs, had a qualified success rate of only 58%, and had a high rate of transient postoperative hypotony.

These early trials informed the final design of the MicroShunt: an 8.5-mm long tube with no endplate having an internal diameter of 70 μm and a pair of fixation fins located distal to the tube’s midpoint, which are intended both to prevent peritubular flow and anchor the device within the sclera to prevent proximal migration into the eye [9, 10]. This design provides controlled filtration of aqueous from the anterior chamber into the subconjunctival/sub-Tenon space when the device is implanted via an ab externo approach. In a second study in the Dominican Republic (DR II), 23 surgically naïve eyes uncontrolled on maximal medical therapy underwent implantation with adjunctive MMC 0.4 mg/mL for 3 min; an additional surgical modification was the creation of a 1 × 1 mm sclera pocket at the distal end of the scleral insertion tract to receive the newly-added fins. At 1 year, these devices and procedural modifications resulted in a qualified success rate of 100%; a reduction in IOP from 23.8 ± 5.3 to 10.7 ± 2.8 mmHg; and a reduction in medication use, from 2.4 ± 1.0 to 0.3 ± 0.8. Only two eyes experienced transient hypotony, both of which resolved with conservative management [10].

These encouraging results laid the foundation for further clinical development of the MicroShunt.

### Clinical data

The efficacy and safety of the MicroShunt have been characterized in several case series and cohort studies spanning up to 5 years and in one large, randomized control trial. A two-center, two-site study in France and the Dominican Republic implanted the MicroShunt into 87 eyes with primary open-angle glaucoma (POAG) on maximally tolerated medical therapy. Mean IOP reductions of 38–55% and medication reductions of 72–88% were achieved at 1 year, with the best outcomes (55% IOP reduction and 85% medication reduction) achieved when 0.4 mg/mL MMC (on pledgets) was utilized [12]. No sight-threatening adverse events were observed.

The 23 eyes that underwent MicroShunt implantation with the higher dose of MMC (0.4 mg/mL) were followed for an additional 5 years [13, 14]. The reductions in IOP and number of medications observed after 1 year were maintained during years 2 through 5. At years 4 and 5, 78.3% and 82.6% of eyes, respectively, attained IOP ≤ 14 mmHg, and 78.6% and 61.1%, respectively, were medication-free. Most ocular adverse events observed through 5 years were transient and self-limited. Serious procedure- or device-related adverse events included two eyes each with posterior capsule opacification, choroidal effusion/detachment, and exposed Tenon’s capsule; however, only two eyes required reoperation for bleb failure.

A study of eight eyes of seven Japanese patients with POAG followed up for a mean 68.9 ± 9.7 months found that IOP was significantly lower at years 2 through 5 than at baseline (all *P* values less than 0.05) and that the mean number of medications was reduced from 3.5 at baseline to 1.5–1.6 at years 1 through 4 [15].

The effects of standalone MicroShunt implantation were retrospectively assessed in 164 eyes of 132 patients with uncontrolled and/or progressing open-angle glaucoma despite maximal medical therapy [16]. The primary outcome measures, IOP 6–17 mmHg and IOP reduction ≥ 20% without medications (complete success) or with medications (qualified success), were achieved in 76.9% and 92.5% of eyes, respectively. Mean IOP decreased from 21.4 mmHg at baseline to 13.3 mmHg at 12 months, and mean number of medications decreased from 3.4 to 0.5. Needling was performed in 14 eyes, surgical revision in two, and reoperation for failure in one.

A retrospective cohort study assessed 1-year outcomes of MicroShunt implantation plus MMC in 85 eyes of 79 patients with IOP above target that were refractory to previous subconjunctival filtering surgery [17]. Median IOP was reduced from 22.0 to 13.0 mmHg and median number of medications from 4 to 0. The rates of complete and qualified success, defined as described above, were 61.0% and 79.7%, respectively. Ten eyes (11.8%) underwent needling, 7 (8.2%) underwent anterior chamber reformation, and 6 (7.1%) underwent reoperation.

An ongoing prospective, randomized clinical trial compared MicroShunt implantation with trabeculectomy in 527 eyes of 527 patients in the United States with mild to severe POAG with IOP inadequately controlled on maximal tolerated medical therapy [18]. Patients aged 40–85 years with IOP ≥ 15 and ≤ 40 mmHg were randomized 3:1 to undergo standalone MicroShunt implantation (n = 395) or trabeculectomy (n = 132), with MMC 0.2 mg/mL applied to all eyes for 2 min and followed up for 2 years. Interim analysis at 1 year showed that the rate of success, defined as a ≥ 20% reduction in baseline IOP without increasing the number of medications, was significantly lower in the MicroShunt than in the trabeculectomy group (53.9% vs. 72.7%, *P* < 0.01). Mean IOPs at 1 year in these two groups were reduced 29.1% and 45.4%, respectively (*P* < 0.01 each), with the mean numbers of medications showing similar reductions, with 71.6% and 84.8%, respectively, being medication-free. Of these patients, 40.8% and 67.4%, respectively, required postoperative interventions, including laser suture lysis (*P* < 0.01); and 28.9% and 49.6%, respectively, experienced transient hypotony (*P* < 0.01). The rate of bleb leak was lower in the MicroShunt than in the trabeculectomy group (8.9% vs. 14.5%), although the difference was not statistically significant. Vision-threatening complications were reported in 1.0% and 0.8% of patients in the MicroShunt and trabeculectomy groups, respectively [18].

At 2 years, the success rate was lower in the MicroShunt (50.6%) group than in the trabeculectomy (64.4%) group [19]. The mean IOP in the MicroShunt group was reduced from 21.1 mmHg at baseline to 13.9 mmHg at 2 years (*P* < 0.01), and the mean number of medications was reduced from 3.1 at baseline to 0.9 at 2 years. In the trabeculectomy group, mean IOP was reduced from 21.1 mmHg at baseline to 10.7 mmHg at 2 years (*P* < 0.01) and the mean number of medications from 2.9 to 0.4. Glaucoma reoperation rates were 18.7% in the MicroShunt and 10.6% in the trabeculectomy group (*P* = 0.01), whereas the rates of hypotony, defined as IOP less than 6 mmHg on two consecutive visits, were 3.8% and 15.2%, respectively (*P* < 0.01). Few serious postoperative complications were reported between years 1 and 2 in either group.

A prospective 2-year, multicenter, single-arm study evaluated MicroShunt implantation (with 0.2 or 0.4 mg/mL MMC for 2–3 min) in 107 eyes of 107 patients with IOP ≥ 18 and ≤ 35 mmHg [20]. The 81 eyes in the per-protocol population showed a decrease in IOP from 21.7 ± 3.4 to 14.5 ± 4.6 mmHg at 1 year and 14.1 ± 3.2 mmHg at 2 years (*P* < 0.0001). The mean number of medications decreased from 2.1 ± 1.3 at baseline to 0.5 ± 0.9 at 2 years (*P* < 0.0001), with 73.8% of patients being medication free. Of the patients treated with 0.2 and 0.4 mg/mL MCC, 51.9% and 90.3%, respectively, were medication free (*P* = 0.001). The most frequent non-serious adverse events were increased IOP requiring medication or trabeculoplasty (25.9%) and mild-to-moderate keratitis (11.1%). Six eyes (7.4%) required reoperations and 5 (6.2%) required needling. No long-term sight-threatening adverse events were reported.

Table 1 summarizes the results of clinical studies of the MicroShunt.

**Table 1:** Results of selected clinical studies of the MicroShunt device

Author, year	Study design	Follow-up (years)	No. of eyes/no. of patients	MMC dose (mg/mL)	IOP (mmHg)^a^		No. of medications^a^	
					Baseline	At follow-up	Baseline	At follow-up
Riss et al. 2015 [12]	Retrospective, two-center, two-surgeon	1	23/23	0.4 near limbus	23.8 ± 5.3	10.7 ± 2.8	2.4 ± 0.9	0.3 ± 0.8
			31/31	0.2 near limbus	27.9 ± 6.7	13.3 ± 3.3	2.5 ± 1.4	0.5 ± 1.0
			33/33	0.4 deep in pocket	25.4 ± 7.9	15.7 ± 4.6	2.9 ± 1.0	0.8 ± 1.3
Batlle et al. 2016 [13]	Single-center case series	3	23/23	0.4	23.8 ± 5.3	10.7 ± 3.5	2.4 ± 0.9	0.7 ± 1.1
Batlle et al. 2021 [14]	Single-center case series	5	23/23	0.4	23.8 ± 5.3	12.4 ± 6.5	2.4 ± 1.0	0.8 ± 1.3
Ahmed et al. 2022 [15]	Single-center, non-randomized	6	8/7	Not stated	17.9 ± 3.5	13.5 ± 3.1	3.5 ± 0.5	2.0 ± 1.1
Schlenker et al. 2020 [16]	Retrospective case series	1	164/132	0.2–0.5	20 (IQR 16.5–26)	12 (IQR 10–15)	4 (IQR 3–4)	0 (IQR 0–0)
Durr et al. 2022 [17]	Retrospective cohort	1	85/79	Not stated	22 (IQR 18–29)	13 (IQR 10–17)	4 (IQR 3–4)	0 (IQR 0–2)
Baker et al. 2021 [18]	Prospective randomized controlled trial	1	MicroShunt group 395/395	0.2	21.1 ± 4.9	14.3 ± 4.3	3.1 ± 1.0	0.6 ± 1.1
			Trabeculectomy group 132/132	0.2	21.1 ± 5.0	11.1 ± 4.3	3.0 ± 0.9	0.3 ± 0.9
Panarelli et al. 2021 [19]	Prospective randomized controlled trial	2	MicroShunt group 395/395	0.2	21.1 ± 4.9	13.9 ± 3.9	3.1 ± 1.0	0.9 ± 1.3
			Trabeculectomy group 132/132	0.2	21.1 ± 5.0	10.7 ± 3.7	2.9 ± 0.9	0.4 ± 0.9
Beckers et al. 2022 [20]	Prospective, multi-center, single-arm	2	81/81	0.2–0.4	21.7 ± 3.4	14.1 ± 3.2	2.1 ± 1.3	0.5 ± 0.9

*IQR *= interquartile range; *IOP* = intraocular pressure; *MMC* = mitomycin C

^a^Mean ± standard deviation values reported, except for Schlenker et al. (2020) and Durr et al. (2022) which report median values

### Surgical technique

The MicroShunt is implanted via an ab externo approach, and ideally placed in one of the superior quadrants. A traction suture may be placed superiorly in the peripheral cornea for downward rotation of the globe to enhance visualization of the surgical field. Anesthesia is accomplished with topical tetracaine 1% and preservative-free lidocaine 1% injected under the dissected conjunctival/Tenon’s flap. Following the creation of a small (approx. 3 clock-hour) conjunctival peritomy, blunt and sharp dissection are employed as needed to create a deep sub-Tenon’s pocket between the medial and superior recti muscles. Cautery is applied as necessary, and MMC is then applied via sponge throughout the pocket. Following irrigation to remove residual MMC, a microknife is used to make a 1-mm wide scleral tunnel beginning 3 mm posterior to the limbus. A 25-gauge needle is inserted into the anterior chamber through the trabecular meshwork at the level of the iris plane. The device is then implanted into the scleral tunnel with its wings seated in the winged incision. Patency and flow through the device are confirmed via irrigation of balanced salt solution into the anterior chamber via a paracentesis. Two-layer closure of the conjunctiva and Tenon’s layer is then performed, paying attention to avoid catching the device’s distal tip within Tenon’s layer.

In comparison to standard trabeculectomy technique, this procedure obviates the need for formation of a partial thickness scleral flap, sclerostomy, iridectomy, and scleral suturing. These key differences can reduce both the surgical time and the intraoperative complication rate. Furthermore, as there is only a 25-gauge entry into the anterior chamber, there was no anterior chamber shallowing or resultant potential intraoperative complications. Another key difference between trabeculectomy and MicroShunt surgery is the location of aqueous outflow which determines the location of bleb formation. In trabeculectomy, the scleral flap, under which aqueous percolates extends to the limbus, permits anterior flow with bleb formation close to the limbus, although some surgeons do not extend sclera incisions to the limbus to minimize anterior filtration. With the MicroShunt, however, aqueous is delivered at least approximately 6 mm posterior to the limbus and is directed posteriorly upon egress from the device. This difference is important given that anterior filtration is associated with elevated cystic blebs at higher risk of leak and other bleb-related complications, while posterior filtration tends to produce lower, more diffuse blebs with lower complication rates [21].

### The role of MMC

From the earliest days of trabeculectomy, the detrimental effects of postoperative fibrosis and scarring were recognized as a common cause of surgical failure. Wound modulation using the antifibrotic agent MMC has been found to improve long-term surgical outcomes [22]. Similarly, MMC is essential for the long-term success of novel subconjunctival filtering procedures including both the MicroShunt and the gel stent [12, 23]. In addition, MMC injection allows determination of the amount delivered to each eye. Early studies suggested that the concentration of MMC was of minimal importance for surgical success [24–26], but subsequent reports have demonstrated benefit at a higher dose of MMC, typically 0.4 mg/mL [27–29].

Several studies have sought to clarify the optimal use of MMC—dose, exposure time, route of administration, and placement—for implantation of the MicroShunt. Data from various studies suggest that lower dose is less effective than higher dose MMC, with the use of MMC 0.2 mg/mL instead of 0.4 mg/mL being a risk factor for surgical failure [16, 20]. In terms of optimal location for MMC exposure, posterior exposure deep within the sub-Tenon’s pocket provided smaller IOP and medication reductions than more anterior placement [12]. Exposure time is typically 2–3 min and can be calibrated to the number and severity of risk factors for surgical failure.

Regarding the route of administration of MMC, most studies to date have employed sponges or similar absorptive materials to directly apply MMC to the scleral bed after dissection of Tenon’s capsule, as is traditional in trabeculectomy surgery. The delivery of MMC to the scleral bed via subconjunctival/sub-Tenon’s injection has been described [30–35]. Several studies of trabeculectomy and other filtering surgeries have demonstrated similar clinical outcomes and bleb morphologies using either directly sponge-applied or injected MMC [30–35]. Subconjunctival injection of MMC at the beginning of the procedure may reduce surgical time by eliminating the exposure time for sponge-applied MMC and eliminate the risk of retained sponge fragments [13].

### Patient selection and the role of MicroShunt in glaucoma management

Novel minimally invasive subconjunctival filtering procedures such as the MicroShunt fill an unmet need in surgical glaucoma management. MIGS procedures directed at the trabecular meshwork provide modest efficacy in IOP and/or medication reductions with good safety profiles; as such, they are generally indicated for patients with mild-to-moderate POAG who do not require very low target IOP [4–7]. At the other end of the spectrum are traditional trabeculectomy and tube-shunt procedures which deliver substantial IOP and medication reductions but with less favorable safety profiles [2, 3]; accordingly, these procedures are typically reserved for patients with advanced glaucoma recalcitrant to more conservative therapies.

The MicroShunt and gel stent bridge the gap between MIGS and traditional filtering procedures. Results of retrospective studies suggested that MicroShunt effectively reduces IOP [12–17], and these initial findings are now supported by an ongoing prospective, randomized clinical trial in 527 patients. In the first year, the MicroShunt lowered IOP less than trabeculectomy (29.1% vs. 45.4%) but delivered mean IOP of 14 mmHg at 1 year (vs. 11 mmHg with trabeculectomy) with substantially fewer cases of postoperative hypotony (28.9% vs. 49.6%) [18]. Preliminary results indicate these IOP reductions are maintained at 2 years, with few serious postoperative complications [19]. This efficacy/safety balance suggests that the MicroShunt provides good outcomes in eyes with moderate to severe glaucoma that may require greater IOP reduction than would be expected from trabecular MIGS procedures. As seen with further experience, optimization of surgical technique, and higher concentration of MMC, efficacy results are likely to improve beyond what was found in the pivotal randomized controlled trial.

## Conclusion

The glaucoma surgery paradigm is evolving. Novel procedures seek to deliver trabeculectomy-like efficacy in a safer and more controlled fashion. Trabecular procedures offer excellent safety but only moderate efficacy and are best suited for eyes with mild to moderate disease with modest IOP and/or medication reduction goals. For eyes requiring significant IOP reductions and/or low target IOP not achievable on maximal medical therapy, subconjunctival filtration procedures are typically necessary. Novel subconjunctival procedures—the MicroShunt and the gel stent—offer the ability to achieve substantial IOP reductions with potentially less risk than filtering surgery trabeculectomy. As with trabeculectomy, the use of MMC to modulate fibrosis and scarring postoperatively is essential to surgical success, and higher doses (0.4 mg/mL) are more effective than lower doses. Preliminary data from a well-designed and adequately powered clinical trial provides early evidence that the MicroShunt can bridge the gap between trabecular MIGS procedures and filtering surgery in eyes with moderate to advanced POAG; longer-term data will further clarify the role of controlled micro-incisional device-assisted ab externo glaucoma filtering surgery in long-term glaucoma management.

## Data Availability

Not applicable.
